# Relation of left atrial peak systolic strain with left ventricular diastolic dysfunction and brain natriuretic peptide level in patients presenting with ST-elevation myocardial infarction

**DOI:** 10.1186/1476-7120-11-24

**Published:** 2013-07-05

**Authors:** Cem Dogan, Nihal Ozdemir, Suzan Hatipoglu, Ruken Bengi Bakal, Mehmet Onur Omaygenc, Baris Dindar, Ozkan Candan, Mehmet Yunus Emiroglu, Cihangir Kaymaz

**Affiliations:** 1 Kosuyolu Heart & Research Hospital, Cardiology Clinic, Istanbul, Kartal 34846, Turkey

**Keywords:** Acute myocardial infarction, Left atrial strain, Brain natriuretic peptide, Diastolic dysfunction

## Abstract

**Background:**

In patients presenting with ST-elevation myocardial infarction (STEMI), we investigated the relation of left atrial (LA) deformational parameters evaluated by two-dimensional speckle tracking imaging (2D-STI) with conventional echocardiographic diastolic dysfunction parameters and B-type natriuretic peptide (BNP) level.

**Methods:**

Ninety STEMI patients who were treated with primary percutaneous coronary intervention (PCI) and 22 healthy control subjects were enrolled. STEMI patients had echocardiographic examination 48 hours after the PCI procedure and venous blood samples were drawn simultaneously. In addition to conventional echocardiographic parameters, LA strain curves were obtained for each patient. Average peak LA strain values during left ventricular (LV) systole (LAs-strain) were measured.

**Results:**

BNP values were higher in MI patients compared to controls. Mean LAs-strain in control group was higher than MI group (30.6 ± 5.6% vs. 21.6 ± 6.6%; p = 0.001). LAs-strain had significant correlation with LVEF (r = 0.51, p = 0.001), also significant inverse correlations between LAs-strain and BNP level (r = −0.41, p = 0.001), E/Em (r = −0.30, p = 0.001), LA maximal volume (r = −0.41, p = 0.001), LA minimal volume (r = −0.50, p = 0.001) and LV end systolic volume (r = −0.37, p = 0.001) were detected. The cut off value of LAs-strain to predict BNP > 100 pg/ml was determined as 19.9% with 55.3% sensitivity and 77.2% specificity (p < 0.05 AUC:0.7).

**Conclusion:**

Our study showed that LAs-strain values decreased consistently with deteriorating systolic and diastolic function in STEMI patients treated with primary PCI. LA-s strain measurements may be helpful as a complimentary method to evaluate diastolic function in this patient population.

## Introduction

Acute myocardial infarction (MI) results in left ventricular (LV) both systolic and diastolic dysfunction in survivors. During the early phases of MI 38% of patients have impaired relaxation and 24% of patients have restrictive LV filling pattern [1]. The important consequence of diastolic dysfunction is elevated filling pressures [2,3]. LV diastolic dysfunction was related to morbidity and death independently from systolic function in acute MI [4,5]. Thus, assessment of diastolic function and LV filling pressures after MI has important prognostic implications.

Diastolic function can be evaluated with several non-invasive and invasive techniques [3]. Tissue Doppler imaging (TDI) derived indices, including systolic velocity (S), early (Em) and late (Am) diastolic velocities of mitral annulus and early mitral inflow peak velocity (E)/Em ratio are sensitive and widely used parameters to estimate LV filling pressures [6]. Increased B-type natriuretic peptide (BNP) levels also provide reliable estimation of LV filling pressures, especially for left ventricular end diastolic pressure (LVEDP) and pulmonary capillary wedge pressure (PCWP) [7-9].

Left atrial (LA) function and morphology is affected by increased LV filling pressures. Assessment of LA strain using two dimensional speckle tracking imaging (2D- STI) is a recently introduced and accurate method for evaluating LA functions. Recent studies have shown clinical importance of LA strain in atrial fibrillation and cardiomyopathy [10,11]. Moreover, decreased LA peak strain during LV systole (LAs-strain) was related to increased LVEDP, previously [12,13].

In the setting of ST-elevation myocardial infarction (STEMI) treated with primary percutaneous coronary intervention (PCI), we aimed to investigate the effects of diastolic dysfunction detected by echocardiography and BNP on LA deformational parameters evaluated with 2D-STI. We also measured phasic LA volumes and assessed their relation to diastolic dysfunction and LA strain.

## Methods

### Study population

Study population consisted of 90 (47 anterior, 43 inferior) consecutive STEMI patients who were treated with primary PCI in our institution and 22 healthy subjects (with no known history of cardiovascular disease, hypertension and diabetes mellitus) as a control group. To constitute the study population 150 consecutive STEMI patients were examined and 90 of them fulfilled the inclusion criteria for the MI group which were; single vessel disease, patients with no known history of previous cardiovascular disease, definite diagnosis of STEMI, successful treatment with primary PCI with restoration of TIMI flow grade 2 or 3 and absence of cardiogenic shock. Patients with atrial fibrillation, moderate-to-severe valvular stenosis or regurgitation, as well as patients whose LA had an insufficient imaging quality, were also excluded from the study. The study was approved by the local ethical committee (Institutional Review Board: Kosuyolu Heart Center İnstutitional Board). Oral and written informed consent was obtained from the patients.

### Risk factors

Arterial hypertension was defined as a reported blood pressure of >140/90 mm Hg or in patients receiving anti-hypertensive therapy. Diabetes mellitus was defined according to the World Health Organization definition as a fasting blood glucose concentration of >126 or >200 mg/dL 2 h after an oral glucose tolerance test or in patients receiving permanent medical anti-diabetic therapy. Hyperlipidemia was defined as blood total cholesterol concentrations of >180 mg/dL or low density lipoprotein of >130 mg/dL or when patients were receiving permanent treatment with lipid-lowering agents.

### Conventional echocardiography

All patients underwent an echocardiographic examination in the left lateral position, using the GE Vivid 7 system (GE Vingmed Ultrasound AS, Horten, Norway) with a 3.5 MHz transducer. Blood pressure and heart rate were continuously monitored during the transthoracic examination. All data were transferred to a workstation for further offline analysis (EchoPAC PC; GE). STEMI group patients had echocardiographic examination 48 hours after the PCI procedure. Transmitral early (E) and atrial contraction (A) flow velocities were obtained by pulsed-wave Doppler in the apical four-chamber view. The ratio of E/A velocity and E-wave deceleration time were measured. For TDI, the same echocardiography machine was used to acquire TDI data at high frame rates. The Nyquist limit was set at 15–20 cm/s, and minimal optimal gain was used. The myocardial systolic (Sm), early diastolic (Em), and late diastolic (Am) velocities were obtained at the septal and lateral mitral annulus by placing a tissue Doppler sample volume. The E/Em for septal and lateral annulus, additionally E/A ratios were subsequently calculated. LA dimensions and LV end-systolic (LVESD) and end-diastolic diameters (LVEDD) were measured. LV ejection fraction (LVEF) was estimated by Simpson's biplane method using apical 4-chamber and 2-chamber views.

The following LA volumes were measured from apical 4 and 2 chamber views by using biplane modified Simpson’s method and indexed to body surface area (BSA):

Maximum LA volume (LAV_max_): measured during LV contraction just before mitral valve opening

Minimum LA volume (LAV_min_): measured at the QRS onset on electrocardiography

LA reservoir volume (LAV_res_): LAV_max_ − LAV_min_

LA ejection fraction (LAEF): [LAV_max_] − [LAV_min_]/[LAV_max_]

### 2-D speckle tracking imaging analysis of left atrium

**Figure 1:**
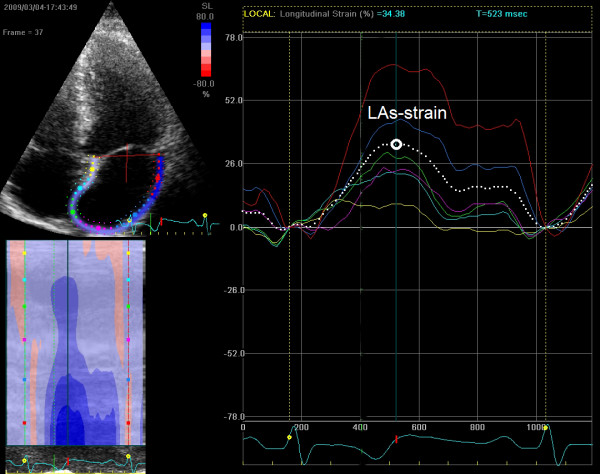
**Measurement of LAs-strain on an image obtained from apical four-chamber view.***LAs-strain, peak left atrial strain during left ventricular systole.*

Two-dimensional echocardiography images for the LA were obtained from the apical four-chamber view. All images were obtained while the patients held their breath at end expiration and the images were stored in a cine-loop format from three consecutive beats. The frame rate for images was set between 60 and 90 frames/s. After defining the endocardial border manually, tracings were developed by the software system automatically for each frame. If the automatically obtained tracking segments were adequate for analysis, the software system was allowed to read the data, whereas analytically inadequate tracking segments were either corrected manually or excluded from the analysis. Image tracking algorithm divided the LA wall into 6 segments automatically. The most frequently excluded segments were the ones containing pulmonary vein entrances and LA apex. The typical LA strain curves were obtained for each patient. Average peak LA strain values during LV systole (LAs-strain) for 6 segments were measured (Figure 1).

### BNP measurement

Venous blood samples were drawn simultaneously with echocardiographic examination both in STEMI patients and control subjects. The samples were immediately centrifuged at 1500 rpm for five minutes and plasma was stored at -80C° until the time of measurement. The quantitative determination of BNP in plasma was done by immune fluorescence method using the ADVIA Centaur System (Bayer Health Care, Tarrytown, NY, ABD).

### Reproducibility

Intra- and inter-observer reproducibility for the LAs-strain was assessed. For intra-observer assessment, images from randomly selected 25 patients were re-analyzed after a week. The Bland–Altman analysis and the intra-class correlation coefficient (ICC) showed good inter- and intra-observer agreement; for inter-observer agreement for LAs-strain, the mean difference was 1.8 (−1.9, 4.5) and ICC 0.87; and for intra-observer agreement, the mean difference was 1.3 (−2.1, 3.2) and ICC 0.91.

### Statistical analysis

Continuous variables were expressed as mean (SD) or median (interquartile range) as appropriate. The level of significance was 0.05. To compare parametric continuous variables, the independent Student t-test or the Mann–Whitney U-test was used. For categorical variables, the x^2^ test was used. Correlations between variables were tested by using the Pearson or Spearman’s correlation tests. Receiver-operating characteristic (ROC) curves were plotted to determine the optimal cut-off values for LA strain of STEMI patients in order to predict increased BNP. Statistical analyses were performed mainly using SPSS, version 15.0 for Windows; and Medcalc software for Windows was also used when necessary.

## Results

There was a male dominancy in both MI (%89) and control (%83) patients; the mean age of subjects were 52.4 ± 8.7 years in MI group and 50.1 ± 4.7 years in control group. Demographic characteristics of STEMI patients are shown in Table 1, also comparison of some clinical characteristics of STEMI and control patients are presented in Table 2. Average time spent for offline analysis of LAs-strain for each patient was 4 minutes and %94 of LA segments were tracked appropriately by the software and included in our analysis.

**Table 1:** Demographic characteristics of STEMI patients

	
Gender, Male (%)	89.4
Age, years (Mean ± SD)	52.4 ± 8.7
Family history (%)	11
Hypertension (%)	30.4
Diabetes mellitus (%)	14.3
Hyperlipidemia (%)	28
Smoking (%)	72
Door to balloon time, min (Mean ± SD)	114 ± 15
MI localization, anterior wall (%)	42.5
Peak CK (mg/dl) (Mean ± SD)	2311.1 ± 1612.5
Peak CK-MB (mg/dl) (Mean ± SD)	222.5 ±158.8
Peak Troponin I (ng/dl) (Mean ± SD)	67.7 ± 42.8

*MI* myocardial infarction.

**Table 2:** Comparison of some clinical characteristics of STEMI and control patients

	**MI**	**CONTROL**	**Ρ**
Age, years	52.4 ± 8.7	50.1 ± 4.7	0.220
Sex, % (Male)	89	83	0.543
BNP, pg/ml*	140 [12–1249]	5 [3-23]	0.001
E, m/s	0.6 ± 0.1	0.8 ± 0.1	0.001
A, m/s	0.7 ± 0.1	0.8 ± 0.1	0.001
E/A	0.8 ± 0.3	1.4 ± 0.3	0.001
Em septal, cm /s	5.7 ± 1.7	7.2 ± 1.5	0.001
Em lateral, cm/s	6.3 ± 1.9	8.0 ±1.8	0.001
E/Em septal	13.6 ± 3.1	11.0 ± 1.9	0.001
E/Em lateral	13.2 ± 2.7	10.8 ± 1.4	0.001
LVEDV, ml	110.1 ± 18.4	107.0 ± 18.6	0.5
LVESV, ml	59.4 ± 15.2	41.7 ± 8.1	0.005
LVEF, %	46.6 ± 9.2	61.0 ± 5.2	0.001

*BNP* indicates brain natriuretic peptide, *E* early mitral inflow velocity, *A* mitral inflow velocity during atrial contraction, *Em* early mitral annular tissue Doppler diastolic velocity, *LVEDV*, left ventricular end diastolic volume, *LVESV* left ventricular end systolic volume, *LVEF* left ventricular ejection fraction.

*Median values and range are given in the table for BNP.

BNP values were significantly higher in MI patients compared to control subjects (140 [12.0-1249.5] vs. 5.0 [3.0-23.1]; pg/ml p = 0.001). The differences between the measurements of transmitral flow velocities (E, A) and E/Em, E/A ratios of the groups were also significant (See Table 2). Maximum and minimum left atrial volumes were increased in MI patients (Table 3). Mean LAs-strain in control group was higher than MI group (30.6 ± 5.6% vs. 21.6 ± 6.6% p = 0.001).

**Table 3:** Echocardiographic assessment of left atrium

	**MI**	**CONTROL**	**Ρ**
LAV _max_ (ml)	47.6 ± 12.5	41.1 ±10.4	0.001
LAV _min_ (ml)	23.2 ± 16.3	16.3 ± 5.6	0.001
LAV _res_ (ml)	26.1 ± 8.7	21.7 ± 5.3	0.010
LA-s strain (%)	21.6 ± 6.6	30.6 ± 5.6	0.001
LAEF (%)	51.7 ± 8.3	60.4 ± 10.3	0.001

*LAV* indicates left atrial volume index, *LA* left atrium, *LAs-s strain* peak left atrial strain during left ventricular systole, *max* maximal; *min* minimal, *res* reservoir.

**Figure 2:**
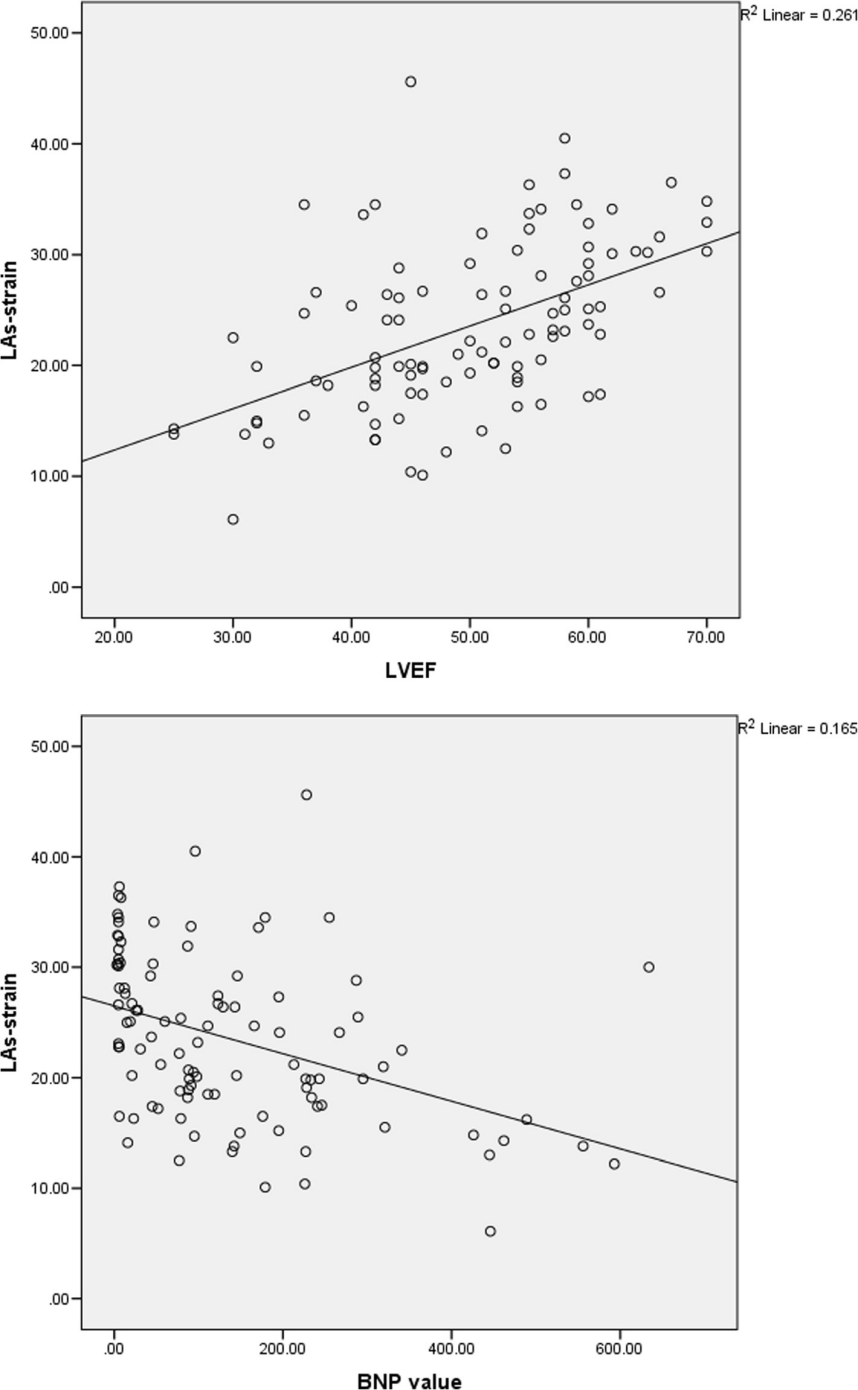
**Correlations of peak left atrial strain during left ventricular systole with BNP level and LVEF.***BNP, brain natriuretic peptide; LAs-strain, peak left atrial strain during left ventricular systole; LVEF, left ventricular ejection fraction.*

LAs-strain had significant correlation with LVEF (r = 0.51, p = 0.001), also there was a significant inverse correlation between LAs-strain and BNP level (r = −0.41, p = 0.001), E/Em (r = −0.30, p = 0.001), LAV_max_ (r = −0.41, p = 0.001), LAV_min_ (r = −0.50, p = 0.001), LAEF (r = 0.38 p = 0.001) and LVESV (r = −0.37, p = 0.001) (Figure 2).

**Figure 3:**
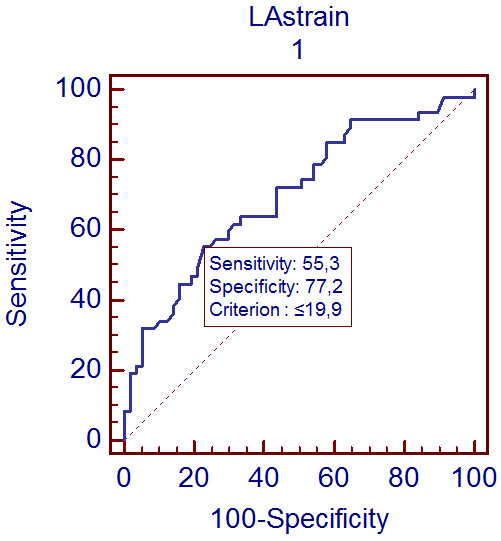
Receiver operating characteristic curve of LAs-strain for the prediction of brain natriuretic peptide level of ≥100 pg/ml. * LAs-strain, peak left atrial strain during left ventricular systole. *The figure was drawn using Medcalc statistical analysis software for Windows.

When the ROC curves were plotted for LA strain to predict increased BNP in STEMI patients; the cut off value of LA strain during LV systole was 19.9% for the MI patients whose BNP values were higher than 100 pg/ml with 55.3% sensitivity and 77.2% specificity (p < 0.05; AUC = 0.7) (Figure 3).

## Discussion

In our study we demonstrated that LAs-strain was significantly reduced after STEMI compared to controls, additionally it was closely related with LVEF and BNP level. E/Em ratio, LA volumes and BNP were also significantly increased in STEMI patients reflecting increased diastoling filling pressures and diastolic dysfunction. Moreover, LAs-strain value of ≤ 19.9% predicted MI patients whose BNP values were higher than 100 pg/ml with 55.3% sensitivity and 77.2% specificity.

Patients who have elevated LV filling pressures after acute MI are more likely to suffer from chronic heart failure and have an increased mortality. Using the E/Em ratio, a close approximation of LV filling pressures can be obtained in a wide spectrum of patients [14,15]. The E/Em ratio is superior to other echocardiographic indices in this respect [16]; and after AMI, an elevated ratio predicts higher mortality and an increased risk of adverse remodeling [17,18]. However, an E/Em ratio between 8 and 15, was associated with a very wide range of mean LV diastolic pressures in the study by Ommen et al. [16]. Because most of our patients had E/Em ratio between 8 and 15, further echocardiographic measurements evaluating LA volumes and function were conducted. E/Em ratios were significantly higher in our STEMI patients.

When indexed to BSA, LA volume is independently associated with increased cardiovascular risk and disease burden [19] and is a powerful predictor of medium-term outcome in patients with suspected heart failure [20], as well as in patients with acute MI [21,22]. We suppose that increased LA volumes measured by Simpson’s method in our STEMI patients was a consequence of LA dilation as an adaptation to LV volume and pressure overload. In the setting of STEMI, initially LA enlargement contributes to improve cardiac output as an adaptive mechanism; however when optimal Frank-Starling relation is exceeded this enlargement results in decreased LA compliance and increased risk of atrial arrhythmias [23].

Two-dimensional speckle tracking imaging was shown in many studies to be useful in quantification of atrial deformation [24,25]. The LA deformation curves obtained with 2D-STI imaging are similar to LA volume curves. LA function and volume curves include three phases known as reservoir, conduit and booster pump [26]. LAs-strain is closely related to the LA reservoir function, besides the LA strain during atrial systole is related to the booster-pump function [12]. The LA reservoir function is assessed in two consecutive phases as early and late. While the early reservoir function depends on LA relaxation, the late reservoir function depends on LV contraction through the descent of the base during systole [27,28]. Therefore, both LA relaxation and LV systolic function might affect the LA reservoir function and so LA-s strain. Wakami et al. investigated the effect of LVEDP on LAs-strain during LV systole [12]. They found that elevated LVEDP is associated with a decrease of LA-s strain. Moreover, several studies demonstrated the superiority of peak systolic atrial myocardial deformation parameters to diastolic atrial deformation parameters, as a predictor of AF and cardiovascular events [29-31]. Thus, in our study we measured LA-s strain in order to assess diastolic dysfunction after STEMI.

In addition to the LAs-strain being an index of the reservoir function, it is also closely related to LV systolic function as mentioned previously [27]. Cameli et al. reported a good correlation between the peak LA strain and PCWP in patients with advanced heart failure [32]. Our findings were also compatible with these data; in our MI patients mean LAs-strain was significantly lower than controls and LA strain was correlated with LVEF. Our MI population exhibited a significant decrease in both systolic and diastolic LV function.

Biomarkers such as BNP may also be used to estimate elevated LV filling pressures [33]. Levels of BNP correlate with increased PCWP and LVEDP [9,34]. We observed that LAs-strain was negatively correlated to BNP level as well as echocardiographic parameters of diastolic function such as E/Em, LAV _max_, LAV _min_ and LV end systolic volume. Additionally, when the cut off value of LA strain during LV systole was accepted to be ≤ 19.9%, MI patients with BNP values ≥100 pg/ml were predicted with 55.3% sensitivity and 77.2% specificity.

The current study has several limitations. Strain rate and strain have been shown to be influenced by preload change and evaluation of LA function by 2D-STI is relatively difficult and time-consuming, besides; to obtain appropriate LA images for strain analysis in patients with insufficient echocardiographic images is difficult and sometimes impossible. The major limitation of our study was that instead of measuring LVEDP invasively, it was estimated using BNP and echocardiographic parameters. The patients were not followed prospectively to assess the prognostic impact of decreased LAs-strain after MI.

In conclusion, we observed that LAs-strain values decreased consistently with deteriorating systolic and diastolic function and decreased LAs-strain values also predicted BNP > 100 pg/ml in STEMI patients treated with primary PCI. With this study we suggest that, LA-s strain measurements may be helpful as a complimentary method to evaluate diastolic function when inconclusive results are obtained by conventional echocardiographic parameters.

## Competing interests

The authors declare that they have no conflicts of interest.

## Authors’ contributions

CK,NO and CD constituted the study design; CD, OC, MYE, RBB, BD and MOO performed the echocardiographic examinations and collected the patient data, SH and CD carried out statistical analysis and drafted the manuscript. All authors read and approved the manuscript.
